# Enhancing Musical Experiences for Cochlear Implant Users: Insights From the 2024 International Fall Cochlear Implant Meeting

**DOI:** 10.1097/ONO.0000000000000070

**Published:** 2025-05-21

**Authors:** Katelyn A. Berg, E. Katelyn Glassman, Nicole T. Jiam, David M. Landsberger, David S. Haynes

**Affiliations:** 1 Department of Otolaryngology—Head and Neck Surgery, Vanderbilt University Medical Center, Nashville, Tennessee; 2 MED-EL Corporation, Durham, North Carolina; 3 Department of Otolaryngology—Head and Neck Surgery, University of California—San Francisco, San Francisco, California; 4 Department of Otolaryngology—Head and Neck Surgery, New York University, New York, New York.

## WHY MUSIC AND COCHLEAR IMPLANTS?

As cochlear implant (CI) professionals, we encounter patients struggling with music perception and appreciation, despite adequate speech understanding. Music is a fundamental aspect of the human experience fostering social connections, aiding emotional processing, and contributing to mental health and well-being (1). While rhythm perception remains largely comparable to normal hearing peers (2), difficulties with CI-mediated pitch, melody, and timbre recognition are well-documented (3). These perceptual limitations can significantly impact music appreciation, with many CI users reporting music sounds worse or unnatural with their CI compared to acoustic hearing (4,5). Some CI users even stop interacting with music altogether postimplantation (5). Music-related outcomes and overall quality of life are positively correlated for CI users, with specific benefits noted for physical health, promotion of positive emotions, reduction of negative emotions, increased self-esteem, and enhanced social interactions (6–8). These findings underscore the critical need for continued research and development in improving music perception and appreciation for CI users, given its significant impact on their overall quality of life.

With the shift to patient-centered care models, clinicians have a responsibility to address the goals that motivate patients to pursue a CI. For many patients, this includes the ability to perceive and enjoy music. Consequently, we must prioritize music as we advance CI technology and develop effective rehabilitation strategies. Therefore, at the 2024 International Fall CI Meeting, experts discussed current challenges and opportunities to maximize music perception and appreciation for CI users. This brief communication summarizes these discussions, organized through a typical patient’s journey through the CI process.

## PREOPERATIVE PATIENT CONSIDERATIONS

Understanding CI candidates’ relationship with music is essential—whether they are casual listeners, enthusiasts, or professionals—as their needs may vary significantly. When music plays a large role in a patient’s life, they may delay audiological care for fear of losing their current acoustic hearing or sound quality. This means that the utilization of CIs in professional musicians and music enthusiasts may be even lower than the already low CI adoption rates of 4%–6% for adults in the United States (9,10).

To improve adoption, clinicians can work to build relationships with musicians and music enthusiasts even before they experience disabling hearing loss. One pathway is to establish connections with music organizations such as the Association of Adult Musicians with Hearing Loss and the National Hearing Conservation Association’s Music and Hearing Loss Task Force to educate musicians about ways to protect and monitor their hearing, and to create pathways for referrals to audiology and CI clinics. Clinicians can also work with patient groups and device manufacturers to create peer-to-peer relationships between CI candidates and existing CI users. These peer relationships provide realistic insights and encouragement for potential recipients.

## PREOPERATIVE COUNSELING AND CLINICIAN CONSIDERATIONS

A critical aspect of the discussion in this population involves balancing the goal of improving speech perception with the desire to maintain residual hearing for music perception and appreciation (11,12). Therefore, defining clear and achievable goals for music perception and appreciation with CIs can help manage expectations and increase patient satisfaction. The counseling approach should also be tailored to the patient’s hearing history, with a differentiation between prelingually and postlingually deafened individuals. Postlingual patients often compare their CI experience to their predeafness music experiences, whereas pre-lingual patients do not have this comparative frame of reference, which might result in greater satisfaction with music with their CI (13). Interestingly, postlingual patients often perform better on music perception tasks than pre-lingual patients, likely due to their prior auditory experience (14).

CI users with any access to acoustic hearing, either through the implanted ear (i.e., electric-acoustic stimulation (EAS) or the nonimplanted ear (i.e., single-sided deaf or bimodal patients) will typically have better music perception and appreciation in their “best aided” listening configuration compared to patients with access to only electric hearing (11,12). Clinicians should ensure patients are fit with an acoustic component to use EAS if unaided threshold at 250 Hz is 80 dB HL or better in the implanted ear (15). For patients with hearing loss in the nonimplanted ear, a hearing aid should be fit, as appropriate, to maximize the use of acoustic hearing.

CI electrode arrays include two main design types (16). Precurved arrays are designed to rest along the inner cochlear wall near the modiolus with the goal of providing more independent channels of information. Straight arrays, on the other hand, are designed to rest along the outer cochlear wall with the goal of providing more cochlear coverage to minimize the spectral upshift in frequency information perceived by the user. For patients with the potential to have preserved low-frequency acoustic hearing, the choice of electrode should prioritize hearing preservation and atraumatic insertion. Despite different electrode designs, music perception outcomes remain similar across electrode types (17). One reason may be that there continues to be variability in where electrode arrays are placed relative to their intended design (18,19). However, postinsertion imaging software such as Oto-pilot and OTOPLAN can be used to obtain electrode placement information (20,21). These software tools can create electrode deactivation plans to reduce channel interaction based on modiolar proximity and to adjust the electrode frequency allocation assignments to reduce frequency mismatch, respectively. Using image-guided programming strategies can lead to improvements in pitch and melody perception with a CI (17,22,23).

## PROGRAMMING CONSIDERATIONS

Programming with the goal of optimizing music presents ongoing challenges, with debates over whether to create separate music programs or use a single program for all listening situations. This discussion is complicated by the recognition that “music” encompasses a wide array of perceptual challenges, each potentially requiring different optimization approaches. Regardless of the acoustic input, setting CI threshold levels to obtain appropriate aided detection thresholds between 20 and 30 dB HL and setting CI upper stimulation levels based on electrically evoked stapedius reflex thresholds is critical to maximizing the patient’s dynamic range (24,25). A separate music program may be appropriate for CI users who have specific listening situations they want to focus on (26). For example, a classical violinist in an orchestra may benefit from optimizing the balance between frequency range and spectral resolution, potentially focusing on the frequency region of the violin (196–2637 Hz), while also maximizing the dynamic range to help in orchestral settings. In contrast, a bassist in an electronic band may require more gain in the lower frequencies but may not require the same dynamic range as the orchestral violinist. Similarly, someone listening to country or pop music may prefer a program that amplifies vocals while reducing accompanying harmonies, whereas someone listening to a symphony might want to preserve all instrumental elements equally. Given these differences, a one-size-fits-all approach may not be sufficient to address the diverse needs of CI users seeking music appreciation.

In these programming sessions, a common challenge is the terminology disconnect between audiologists and musicians, particularly when discussing technical aspects such as frequency versus semitones/musical notes. To address these challenges and provide optimal care, some centers have suggested implementing dedicated “musician’s clinics” within CI centers that could provide specialized programming and/or group sessions to provide comprehensive care for musicians. However, these alternative clinical models raise logistical challenges such as how to accommodate additional or extended appointments. In addition, these types of appointments and services may not fit neatly into existing billing and reimbursement models.

## REHABILITATION STRATEGIES

Rehabilitation strategies play a crucial role in optimizing music perception and appreciation for CI users. CI users are encouraged to begin music listening postimplantation with songs accompanied by visual aids such as music videos or lyrics to provide additional contextual cues, gradually increasing complexity over time as their perception improves (27). Music with a larger emphasis on rhythm, heavier bass, fewer instruments, and prominent vocals in the mix may be easier for CI users to appreciate (28,29). These elements are more common in genres such as R&B, pop, country, and folk music. Using a gradual, structured approach can help CI users build confidence and skills over time (30,31).

Patient engagement is also crucial for successful rehabilitation. Musicians, accustomed to practice, often find CI listening practice seamless and are keen to join research to improve their listening experience with their CI. While this enthusiasm is beneficial for advancing the field, it’s important to acknowledge the potential bias in research participation. Published studies likely reflect “better performers” since musicians, and those focused on perceptual improvements are more likely to volunteer, compared to those who struggle with or have lost interest in music.

To address this, researchers are developing tools for at-home music rehabilitation for CI users, which may help broaden participation and provide more comprehensive data. For assessment, clinicians should consider incorporating the Music-Related Quality of Life questionnaire (32) into their standard clinical protocol. This validated tool provides a comprehensive evaluation of both music perception abilities and engagement, helping clinicians understand patients’ baseline abilities, identify specific challenges, and track progress over time. For rehabilitation, at-home training platforms such as Meludia (https://meludia.com) offer comprehensive music perception training programs (33,34). For pediatric patients, specialized programs such as MED-EL’s Musical Ears (https://academy.medel.com/) and online musical games from Inside the Orchestra (https://insidetheorchestra.org/) provide age-appropriate training activities. Additionally, freely available resources such as AngelSound (https://angelsound.tigerspeech.com/)(35) and TeamHearing (https://teamhearing.org/) offer various exercises focused on different aspects of music perception. These tools allow clinicians to create individualized rehabilitation plans based on patients’ specific needs and goals as identified through initial assessments. Moreover, rehabilitation programs must consider the diverse needs of various patient groups, such as pediatric patients or those with single-sided deafness, as these populations may require distinct strategies to optimize their music perception abilities.

## FUTURE DIRECTIONS

There is increasing focus on improving music perception and appreciation for CI users, yet significant challenges remain. The structure of current clinical sessions and the tools to assess music perception within them are inadequate, being either too time-consuming or lacking sensitivity. More research is needed on how to design ideal clinical models and how to best assess music within the clinic. By advancing devices and patient-centered approaches, CI professionals can provide CI users with richer, more fulfilling music experiences.
